# Applications of functional neurotransmitter release imaging with genetically encoded sensors in psychiatric research

**DOI:** 10.1038/s41386-024-01903-5

**Published:** 2024-06-28

**Authors:** Emily C. Wright, Erin Scott, Lin Tian

**Affiliations:** 1 Max Planck Florida Institute for Neuroscience, Jupiter, FL USA; 2 Neuroscience Graduate Group, University of California, Davis, CA USA; 3 Department of Biochemistry and Molecular Medicine, University of California, Davis, CA USA

**Keywords:** Preclinical research, Neuroscience, Psychiatric disorders

## Abstract

Psychiatric research encompasses diverse methodologies to understand the complex interplay between neurochemistry and behavior in mental health disorders. Despite significant advancements in pharmacological interventions, there remains a critical gap in understanding the precise functional changes underlying psychiatric conditions and the mechanisms of action of therapeutic agents. Genetically encoded sensors have emerged as powerful tools to address these challenges by enabling real-time monitoring of neurochemical dynamics in specific neuronal populations. This prospective explores the utility of neurotransmitter binding genetically encoded sensors in uncovering the nature of neuronal dysregulation underpinning mental illness, assessing the impact of pharmaceutical interventions, and facilitating the discovery of novel treatments.

## Introduction

Psychiatric research is intrinsically interdisciplinary, built on a rich and diverse history of inquiry. It synthesizes knowledge from a variety of methodologies including individual case studies, demographic analyses, human clinical trials, animal testing, cellular studies, computational simulations, and the evaluation of therapeutic interventions. Historically, the nature of mental health disorders has been characterized through changes in patient behavior and the effectiveness of pharmaceutical or other therapeutic interventions were also gauged by behavioral outcomes. However, there remains much that is still unknown about the changes in neurofunction that drive the behavioral changes that characterize psychiatric disorder. To date, most pharmaceutical treatments for treating mental disorders either mimic or block the action of neurotransmitters, and work to correct partially characterized or hypothesized dysfunction in the complex spatiotemporal regulation of brain chemistry. Notably, despite medications having pharmacodynamic properties that act within minutes, their therapeutic effects often take a long time—weeks—to manifest in patients, indicating poorly understood neuromodulatory effects. The lack of knowledge about the functional effects of many of the commonly prescribed pharmaceuticals on neuronal activity presents a grand challenge in psychiatric research.

To fill gaps in knowledge, we need a comprehensive theoretical framework underlying neurotransmitter release, interaction, and integration of these signals. This will allow for a more complete understanding of how vast quantities of information from a multitude of sources are transmitted between neurons to facilitate changes in behavioral output. In recent decades, technological advancements including large-scale neuronal recording and manipulation, high-throughput genomics, molecular genetic manipulation, and behavioral analysis, have enabled a comprehensive investigation into the mechanisms of brain function in both healthy and diseased states. These developments have been instrumental in refining theoretical frameworks and beginning to unravel the mechanistic action of established medications on brain function. Notably, optical methods, particularly when combined with genetically encoded sensors of neural activity, have become broadly utilized technology in modern neuroscience to record neural activity, due to their high molecular and cell-type specificity, and high spatiotemporal resolution within animals that are actively engaged in processes or tasks of interest.

In this context, we examine how genetically encoded sensors can shed light on the neurological underpinnings of mental illness, illustrate the regulatory effects of pharmaceutical interventions on an imbalanced brain, and assist in the development of novel, targeted pharmaceutical treatments.

## Bridging a gap

Communication between neurons in the mammalian nervous system relies on intricate changes in brain chemistry, both at the local and systemic levels. Rapid neurotransmitters like glutamate and gamma-aminobutyric acid (GABA) operate through ligand-gated ion channels and G-protein coupled receptors (GPCRs), influencing the firing rates and excitability of neighboring neurons. Unlike these quick-acting neurotransmitters, neuromodulators predominantly engage with GPCRs, instigating molecular signaling cascades that affect synaptic strength, neuronal excitability, and circuit dynamics over varying timescales, from fractions of a second to several hours. For a detailed understanding of the biochemical, biophysical, and computational mechanisms underlying neurochemical communication, measurements of release dynamics must be precise in time and space, and be molecularly and cellularly specific.

Conventional techniques for measuring brain activity in humans, such as functional magnetic resonance imaging, electroencephalography, transcranial magnetic stimulation, and positron emission tomography, provide valuable non-invasive insight. However, they are constrained by the limitations inherent to human studies and lack the resolution necessary to delineate cell-type and circuit-specific information. Animal models provide a translational approach to psychiatric research that allows for the use of high-resolution functional imaging techniques necessary to understand the true intricacies of neurochemical communication. Work conducted in animal models is not an exact replication of a human psychiatric condition and can never replace the need for clinical research. That is why it is important for the different facets of psychiatric research to each fill their unique niche of understanding to build a more comprehensive picture of the biology behind mental illness.

Advances in analytical chemistry, protein engineering, and optical technologies have led to the development of sensors and probes for real-time neurochemical monitoring in animal models. Microdialysis, for example, involves the insertion of a probe with a semi-permeable membrane to collect and analyze brain interstitial fluid. Fast-scan cyclic voltammetry represents another leap forward, enabling the sensitive detection of electroactive substances with exceptional temporal resolution. However, both microdialysis and fast-scan cyclic voltammetry offer limited spatial or temporal resolution, restricting chronic measurement of neuromodulator release across the full-behavior paradigm in freely behaving animals.

Genetically encoded sensors based on fluorescence proteins have marked a significant advancement for real-time brain chemistry analysis in recent decades. These sensors can be targeted to specific neuronal populations using cell-type specific viral delivery methods. Their fundamental design links ligand-induced conformational changes in the ligand binding domain with fluorescence intensity changes in the reporting domain, such as circularly permutated green fluorescent protein, providing an optical signal of ligand transients. Typically utilizing a single fluorescent protein, ligand binding induces structural changes that result in altered fluorescence intensity. Genetically encoded sensors have now been developed for a range of neurochemicals including glutamate, GABA, dopamine, norepinephrine, serotonin, acetylcholine, ATP, glucose, and various neuropeptides.

Traditionally, genetically encoded sensors have required transgenic lines to target their expression to specific circuits. However, neurotransmitter-binding sensors offer a transgenic-independent specificity that can be utilized in a diverse array of animal models, as demonstrated by studies utilizing GRAB_ACh_ in *Drosophila* [1], GRAB_DA_ in zebrafish [2], or dLight in mandarin voles [3]. Promoters and enhancers can be employed to confer cell-type specificity including neuronal (hSyn1) and glial (GFA), as well as distinct subpopulations such as dopaminergic, excitatory, and inhibitory neuron cell types.

The array of sensors available, alongside diverse imaging techniques equips us with a comprehensive toolkit for dissecting neural circuits, systems, and behaviors. Fiber photometry, when used with these sensors, enables sub-second tracking of calcium and neurochemical changes in freely moving animals, offering insights into the neural basis of innate and learned behaviors, although it does not achieve single-cell resolution. In contrast, one-photon and multi-photon microscopy provide detailed mapping at the cellular or subcellular level, illustrating distinct neuromodulator releases in response to electrical stimuli or behavior. Complimentary advances in machine learning, as exemplified by programs like DeepLabCut [4], have increased the temporal specificity of behavior quantification allowing researchers to achieve near perfect temporal synchrony between brain activity and behavioral output.

## Using genetically encoded sensors to understand brain dysfunction and pharmaceutical impact

Broad applications of these sensors in animal models have not only enabled major new discoveries about fundamental neuromodulator biology but also pathophysiology. Here we highlight a few specific applications of genetically encoded sensors are utilized to better understand the functional activity patterns in preclinical models of psychiatric conditions. We believe these studies serve as compelling examples of how genetically encoded sensors can be effectively applied in preclinical research and serve to open a window into this rapidly growing field.

The glutamate-binding sensor iGluSnFR was essential to examine cortical connectivity changes in a mouse model of depression. Using iGluSnFR, researchers found strength of intracortical connections to increase the depression model and attenuate back to control levels 24 h after ketamine treatment [5]. Discoveries like these allow greater understanding as to how depression state can alter neural circuit function, and also how pharmacological treatment alters these dysfunctional neural states. Similar research is utilizing the ever-growing array of genetically encoded sensors to better understand pathway-specific neurochemical dysregulation in stress and depression models. The dopamine-binding sensor, dLight, has been used show how increased anhedonia in females correlates with decreased dopamine release into dorsomedial striatum [6]. While the serotonin-binding sensor, iSeroSnFR, allowed researchers to understand how early life adversity decreases serotoninergic input from dorsal raphe into orbitofrontal cortex [7]. Additionally, the norepinephrine-binding sensor GRAB_NE_ allowed researchers to observe important differences in a rat line with low endogenous levels of corticosterone, which is hypothesized to be a risk factor for increased stress susceptibility. Low corticosterone rats were found to display increased levels of hippocampal norepinephrine that directly correlates with decreased rapid eye movement (REM) sleep [8]. This finding grants greater insight into how biological variability may lead to increased stress sensitivity and risk for the development of depressive/anxiety disorders.

Not only can genetically encoded sensors be applied to understand the biology of depression, they can also be used to asses safety concerns surrounding new lines of treatment. A growing body of research supports the use of ketamine as a treatment for depression disorders, but concerns remain about its addictive potential. Researchers used the dopamine-binding sensor dLight to compare the effects of ketamine to cocaine, a known addictive substance, on known functional changes associated with addiction, finding compelling evidence that ketamine does not induce the full array of addiction markers seen after cocaine administration [9].

**Fig. 1:**
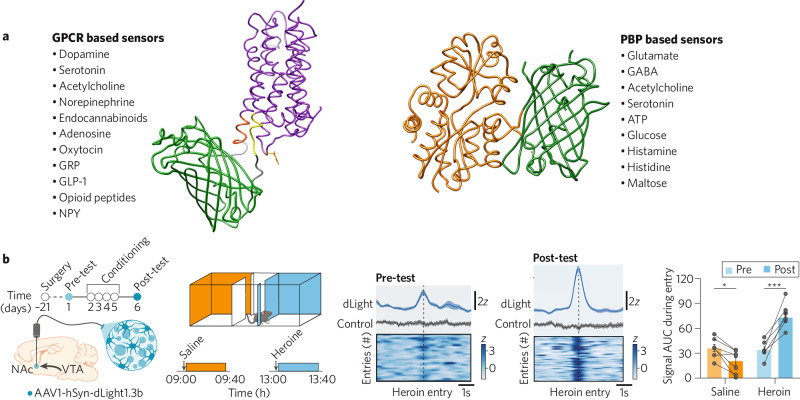
Currently available neurotransmitter binding sensors and preclinical research example. **a** Representative list of currently available GPCR and PBP based neurotransmitter binding sensors. **b** Example of a genetically encoded sensor used to better understand neuronal pattern changes that occur during drug addiction. The dopamine-binding sensor dLight has been used to demonstrate how heroin use changes dopamine-driven reward circuits, increasing reward response upon entry to locations of drug abuse. Adapted from [10].

Genetically encoded sensors are also being applied with great success to better understand the neurobiology of substance abuse disorders. The dopamine-binding sensor dLight has been used to demonstrate how heroin use changes dopamine-driven reward circuits, increasing reward response upon entry to locations of drug abuse [10] (Fig. 1b). Additionally, dLight has been employed to investigate how cocaine usage dysregulates activity in the nucleus accumbens [11]. The dynorphin-binding sensor kLight was able to evaluate how withdrawal from morphine induces endogenous release of dynorphin in prefrontal cortex, a phenomenon known to cause aversion and disrupt cognition [12]. Furthermore, the serotonin-binding sensor GRAB_5HT_ has been used to help understand neural mechanisms of addiction vulnerability. Researchers found serotonin levels in dorsal striatum to be inversely correlated with compulsive cocaine seeking behavior in mice [13]. Together these and other studies aim to understand circuit-level changes that occur during addiction and investigate methods of prevention.

Genetically encoded sensors are also being used to understand the functional activity that underlie hallucination, a symptom that may present during conditions like schizophrenia and bipolar disorder. Both the dopamine-binding sensor GRAB_DA_ and the serotonin binding sensor psychLight have been instrumental in unlocking new information regarding the pathways involved during a hallucinatory event [14, 15].

Recently, the development and implementation of optogenetic tools including genetically encoded sensors have allowed researchers to identify the dysregulated neurotransmission and circuit dynamics underlying neurodegenerative disorders including Parkinson’s disease. The dopamine-binding sensors dLight and GRAB_DA_ allows for high sensitivity detection of dopamine with circuit and cell-type specificity, leading researchers to insights into the mechanisms and patterns of dopamine release deficits along disease progression. Utilizing dLight, researchers found that disruption of mitochondrial complex I (MCI) corresponds with a progressive loss of dopaminergic signaling first in the dorsal striatum, with eventual depletion of dopamine tone in substantia nigra. This staging of dopamine depletion in MCI depleted mice paralleled a cumulative loss in fine motor skill and gross motor deficits, suggesting dopamine depletion in striatum may be necessary but not sufficient to induce clinical parkinsonian phenotypes [16]. Furthermore, using the calcium activity indicator GCaMP alongside the dopamine sensor GRAB_DA_, researchers identified prodromal alpha-synuclein induced degeneration of structural and functional properties of dopamine neurons. These burdens, including increased calcium and dopamine burst firing, preceded neuronal demise. However, as assessed using GRAB_DA_, progressive dysregulation in dopamine signaling could be reversed through pharmacological manipulation with D2 receptor agonists [17]. This suggests neuronal loss may be mitigated and opens avenues for exploration into mechanistic pathways to reveal intersectional treatments for Parkinson’s disease.

## Using genetically encoded sensors to discover new pharmacological treatments

Genetically encoded sensors not only enable the measurement of neural mechanisms underlying mental illness but also hold promise for advancing the discovery of improved pharmaceuticals for psychiatric treatment. As we better understand the mechanistic changes that are characteristics of different mental illness, we can design new pharmaceuticals more specifically targeted to rescue function back to baseline while minimizing off target effects. Due to their ability to selectively target and modulate neural function, drugs targeting GPCRs have become a major focus of this research, with many new candidates undergoing clinical trials. Notably, approximately 35% of all FDA-approved drugs exert their effects through GPCRs [18]. The application of genetically encoded sensors is proving instrumental in assisting researchers in uncovering and evaluating the efficacy of GPCR-targeted pharmaceuticals, helping to contribute to the evolution of a new era in mental health treatment.

A significant portion of genetically encoded sensors use GPCRs as ligand binding domains (Fig. 1a). Consequently, drugs that bind to the endogenous GPCR will also bind to the corresponding GPCR-based sensor as long as the sensor retains the receptor pharmacology. For example, the serotonin-binding sensor psychLight is constructed from the endogenous serotonin-binding GPCR 5-hydroxytryptamine 2 A receptor (5-HT2AR). In addition to serotonin, psychiatrically relevant pharmaceuticals including many hallucinogens, atypical antipsychotics, and psychoplastogens also bind to 5-HT2AR. One such compound, psilocybin, has come to prominence as a treatment for post-traumatic stress disorder [19]. Psilocybin is thought to exert its therapeutic effect by increasing dendritic spine growth and an overall enhancement of neural plasticity [20]. However, it also induces hallucinogenic effects, making psilocybin difficult to prescribe and only able to be administered to patients in a clinical setting. Thus, there is a desire to screen for similar plasticity inducing compounds that do not elicit hallucination. With this goal in mind, researchers used in-vitro expression of psychLight to screen plasticity inducing drugs with unknown hallucinogenic potential. Compounds with high hallucinogenic potential would strongly bind strongly to psychLight inducing a strong fluorescent response. Researchers were able to identify several promising candidate compounds that increase neural plasticity without exhibiting strong 5-HT2AR binding [15]. In this way, genetically encoded sensors allow for more accurate early-stage screening of a compound’s characteristics to better target ideal drug candidates for furthering testing.

## Future directions

There are many considerations when envisioning future challenges to be met while designing the next generation of genetically encoded sensors and associated imaging technology. There are four main features sensor engineers work to optimize: kinetics, brightness, specificity, and spectral array. Genetically encoded sensors generally have fast kinetics and can capture changes in ligand concentration at the millisecond timescale. Iterative optimization is utilized to screen for sensor variants that have faster kinetics. This is characterized by quick capture and release dynamics- allowing the sensors to more closely map the sub second changes in endogenous ligand concentration. Sensor brightness is also important to be able to accurately measure small concentrations of ligand release into a region. This can be achieved by prioritizing a low dissociation constant (k_d_), meaning the sensor will readily bind and increase fluorescence at a lower ligand concentration. Next, sensor specificity is a very important consideration and one that can prove challenging. Sensor specificity is the selectiveness with which the sensor binds only the ligand of interest and no other ligands. GPCR based sensors may particularly struggle with ligand selectivity, as many of the endogenous GPCRs they are based on naturally have some promiscuity in binding. Finally, there is the need for a wider array of spectrally shifted sensors. A toolbox of spectrally distinct sensors will allow for more precise dissection of neurochemical activity, particularly during multiplex imaging to visualize the dynamics of multiple neurotransmitters in the same region. Some currently available examples include red-shifted iGluSnFR [21], red-and yellow-shifted dLight [22], and red-shifted GRAB_DA_ [23]. The development of red and far-red shifted sensors is also important to enhance imaging depth, increase signal-to-noise ratio through the reduction of tissue autofluorescence, and reduce light-induced cytotoxicity. A challenge with multiplex imaging is that many of the commonly used fluorophores do have modest overlaps in spectral excitation. For example, the optimal excitation wavelength for a green fluorescent protein reporter sensor also mildly excites an mCherry reporter sensor. To combat this, researchers should be careful to select sensors whose fluorophores have little to no spectral overlap, or employ imaging techniques with strict optical filtering.

The complexity of mental health disorders arises from multiple causes, involve various dysfunctional mechanisms, and cannot be effectively addressed with a one-size-fits-all treatment strategy. Personalized psychiatric care, which requires a nuanced understanding of the specific disruptions in neural activity and their behavioral implications, is increasingly recognized as essential. This approach aims to precisely rectify the abnormalities that degrade quality of life. Despite the urgent societal need for improved treatments for psychiatric and neurodegenerative conditions, many standard therapies still rely on outdated drugs. The advent of genetically encoded sensors has been transformative, enabling the targeted monitoring of neurochemical release within specific neural circuits. This capability is crucial for investigating neurofunctional alterations in animal models of mental illness and for understanding the immediate effects of pharmaceutical interventions on neurochemical activity. Concurrently, genetically encoded sensors are enhancing in-vitro screening for new drugs. With over 100 identified neurochemicals, the existing genetically encoded sensor toolkit covers only a limited number, highlighting the need for ongoing development of new sensors and an expansion of their color spectrum. Such advancements are vital to fully decode the brain’s language and its relationship to both the development and treatment of mental health disorders.
